# The Predictive role of Neutrophil-to-Lymphocyte Ratio (NLR) and Mean Platelet Volume-to-Lymphocyte Ratio (MPVLR) for Cardiovascular Events in Adult Patients with Acute Heart Failure

**DOI:** 10.1155/2021/6889733

**Published:** 2021-10-11

**Authors:** Teeranan Angkananard, Teeraporn Inthanoo, Suchat Sricholwattana, Nattapun Rattanajaruskul, Arthit Wongsoasu, Worawut Roongsangmanoon

**Affiliations:** ^1^Division of Cardiovascular Medicine, Department of Medicine, Faculty of Medicine, HRH Princess Maha Chakri Sirindhorn Medical Center, Srinakharinwirot University, Nakhon Nayok, Thailand; ^2^Department of Medicine, Faculty of Medicine, HRH Princess Maha Chakri Sirindhorn Medical Center, Srinakharinwirot University, Nakhon Nayok, Thailand

## Abstract

**Introduction:**

The inflammatory response plays a potential role for the pathogenesis and adverse outcomes of heart failure (HF). We aimed to explore the predictive role of baseline neutrophil-to-lymphocyte ratio (NLR) and mean platelet volume-to-lymphocyte ratio (MPVLR) on cardiovascular events (CVEs) in patients hospitalized with acute HF.

**Materials and Methods:**

A retrospective cohort study was conducted in 321 patients with HF between January 2017 and December 2019. The association between their NLR, MPVLR, and combined NLR and MPVLR and CVEs, rehospitalization for HF, in-hospital death, and a composite outcome was explored by survival analysis using a Cox proportional hazard model. They were separately investigated and compared with the area under the receiver operating characteristics curve (AUC).

**Results:**

Up to the end of the 3-year follow-up, 96 (29.9%) had CVEs, 106 (33.0%) died, 62 (19.3%) were rehospitalized with HF, and 21 (6.5%) died during admission. The NLR and MPVLR were significantly associated with CVEs (adjusted HR for NLR ≥ 3.29, 3.11; 95% CI, 1.98-4.89; MPVLR ≥ 8.57, 2.86; 95% CI, 1.87-4.39), readmissions for HF (adjusted HR for NLR ≥ 3.58, 2.70; 95% CI, 1.58-4.61; MPVLR ≥ 6.43, 2.84; 95% CI,1.59-5.07), in-hospital mortality (adjusted HR for NLR ≥ 3.29, 9.54; 95% CI, 2.19-41.40; MPVLR ≥ 8.57, 7.87; 95% CI, 2.56-24.19), and composite outcome (adjusted HR for NLR ≥ 3.32, 4.76; 95% CI, 3.29-6.89; MPVLR ≥ 7.07, 3.64; 95% CI, 2.58-5.15). The AUC of NLR and MPVLR for CVEs were 0.67 (95% CI, 0.61-0.72) and 0.63 (95% CI, 0.58-0.69). Combined NLR and MPVLR increased the AUC to 0.77 (95% CI, 0.72-0.83) with statistical significance.

**Conclusion:**

The elevated NLR and MPVLR on admission in patients with acute HF were independently associated with worse CVEs, rehospitalization for HF, in-hospital death, and composite outcomes. These economical biomarkers should be considered in the management and follow-up care of patients with acute HF.

## 1. Introduction

Heart failure (HF) is a common and serious condition with a global prevalence of 64.3 million [1]. The prevalence is predicted to increase rapidly by 46% from 2012 to 2030 [2]. The 1-year and 5-year mortality rates after HF hospitalization are reported to be as high as 22% and 42.3%, respectively [2]. Therefore, using biomarkers to predict HF outcomes may benefit HF management and reduce the mortality rate.

The inflammatory response plays a potential role in the pathogenesis and adverse remodeling process in patients with both acute and chronic HF [3, 4]. Neutrophil- and T-cell-derived enzymes and cytokines can cause myocardial damage, negative left ventricular remodeling, and disease progression in both HF with reduced ejection fraction and HF with preserved ejection fraction [5]. In addition, platelets are involved in hemostasis, thrombosis, and inflammation and are thus important in disease occurrence. It has been shown that inflammatory processes are regulated by the platelet-induced activation of blood leukocytes, which influences the pathophysiology and progression of chronic HF [6, 7].

The neutrophil-to-lymphocyte ratio (NLR) is an emerging biomarker useful for predicting the risk and prognosis of cardiovascular diseases [8–10]. Thus, its usefulness has been recognized in risk stratification and has been shown to be cost-effective for patients with cardiovascular (CV) disease [11]. Previous studies have demonstrated the relationship between NLR and acute and chronic HF, including their associated complications, severity, and prognosis [12]. A recent meta-analysis [10] also supported the prognostic role of the NLR for all-cause mortality in patients with HF. NLR has a similar prognostic power as the N-terminal probrain B-type natriuretic peptide (NT-Pro, BNP) for major cardiovascular events (CVEs), particularly in elderly patients with HF [13].

Furthermore, the mean platelet volume (MPV), a known biomarker of both proinflammatory and prothrombotic conditions [14, 15], has been found to be an independent variable for predicting in-hospital and 6-month mortality [16] and HF-related hospitalization outcomes in patients with HF [17]. However, all-cause mortality outcomes include both CV and non-CV deaths. No study has thus far investigated the prognostic role of NLR, MPV, MPV to lymphocyte ratio (MPVLR), and combined NLR and MPVLR for a particular outcome of CV mortality and CVEs in patients hospitalized with acute HF (AHF). Hence, we aimed to explore the predictive role of them on CVEs in adult patients with AHF.

## 2. Materials and Methods

### 2.1. Study Design and Data Collection

We conducted a retrospective cohort study using the electronic medical records of consecutive patients with acute HF admitted to the H.R.H Maha Chakri Sirindhorn Medical Center of Srinakharinwirot University between January 1, 2017, and December 31, 2019. Patients included in the study must have been admitted with a principal diagnosis of HF and underwent transthoracic echocardiography. Exclusion criteria included a history of hematologic disease, severe infection, cancer, or recent corticosteroid use within 3 months prior to admission. HF was classified into three groups according to the HF guidelines of the European Society of Cardiology [2]; that is, HF with preserved ejection fraction was defined as left ventricular ejection fraction (LVEF) ≥ 50% while HF with reduced ejection fraction was defined as LVEF < 40%. Patients with LVEF between 40% and 49% were categorized as having HF with midrange ejection fraction.

The primary outcome was a combined CVEs of CV death and hospitalization due to HF; acute coronary syndrome (ACS), including ST-segment elevation myocardial infarction, non-ST elevation myocardial infarction, and unstable angina; acute stroke or cerebrovascular accident; and cardiac arrhythmia. The secondary outcomes included readmission for HF, in-hospital death, and a composite outcome of CVEs and all-cause mortality. Patient data were collected at the index of hospitalization and included demographics, initial vital signs (respiratory rate (RR), pulse rate, and systolic and diastolic blood pressure), New York Heart Association (NYHA) functional classification, concomitant CV diseases, laboratory findings, current medication, electrocardiographic and echocardiogram findings, current medication, clinical course, and length of hospital stay. Clinical outcomes during admission and after discharge were collected from electronic medical records and telephone consults.

NLR was calculated by dividing absolute neutrophil counts by absolute lymphocyte counts derived from automated cell counters. MPVLR was computed by dividing the MPV by absolute lymphocyte counts. Both NLR and MPVLR were classified into two groups using different optimal cut-off values for each outcome. To define the optimal cut-off values of NLR and MPVLR, the receiver operating characteristic curve-based method of the Youden index was used. These cut-off points optimized the differentiating capability of the NLR and MPVLR when an equal weight was given to the sensitivity and specificity [18, 19]. Using data from a previous study [20], the calculation for a sample size revealed that at least 305 patients were needed to achieve a power of 0.8 and a type I error of 0.05.

### 2.2. Statistical Analyses

Continuous variables are expressed as the mean with standard deviation or the median with interquartile range (IQR) and were compared between CVEs and each outcome using the Student *t*-test or Mann-Whitney *U* test, depending on the data distribution. Categorical variables are shown as frequencies and percentages and were compared between groups of outcomes using the chi-square test. Survival probability was assessed using Kaplan–Meier estimation. The relationship between the NLR and MPVLR groups, CVEs, and composite outcomes was compared using a log-rank test. Survival analysis with the Cox proportional hazard model was used to identify factors associated with the NLR and MPVLR groups according to individual outcomes. Hazard ratios (HRs) and their 95% confidence intervals (CIs) were calculated to explore the strength of association between the studied factors (NLR and MPVLR), confounders, and outcomes using Breslow's method for handling ties.

The set of possible confounders for each outcome based on background knowledge and clinical practice was individually considered in the univariate analysis. Variables with a *P* value < 0.1 in the univariate analysis were considered in the multivariate analysis. We excluded the variables that had high missing data (>10%) from the analysis (51.7% magnesium, 57% phosphorus, 42.1% albumin, 50.8% troponin, and 86.3% NT-pro-BNP). The forward selection was used to identify the significant variables for each outcome and the studied factors (NLR and MPVLR). However, MPV was not included in the multivariate model because it was part of our studied factor (MPVLR). The final variables were included in the Cox regression multivariate models for each outcome.

Both NLR and MPVLR were found to have equal ability to predict the risks of our outcomes; thus, we converted them into a combined NLR and MPVLR using a point-based risk score. It was derived from the *β*-coefficients of the final Cox regression multivariate model of individual outcomes (Appendix). We applied categorical NLR and MPVLR for each outcome with different cut-off values (NLR: 3.29, MPVLR: 8.57 for CVEs and in-hospital death outcomes; NLR: 3.58, MPVLR: 6.43 for rehospitalization for HF; and NLR: 3.32, MPVLR: 7.07 for the composite outcome). The predictive abilities of NLR, MPVLR, and combined NLR and MPVLR were separately evaluated and compared in each outcome with respect to the area under the receiver operating characteristic curve (AUC) [21].

All *P* values were two-sided, and a value of less than 0.05 was considered significant. All analyses were performed using STATA version 16.1 (StataCorp; College Station, Texas, United States). The study protocols were reviewed and approved by the Institutional Review Board Committee of Srinakharinwirot University (SWUEC-365/2562E) in April 2020.

## 3. Results

### 3.1. Baseline Characteristics of the Patients with HF Stratified by CVEs

Table 1 shows the demographic, clinical, and laboratory data of the 321 patients with HF. The majority were elderly (62.2%), with a mean age of 67.4 ± 14.9 years, and nearly half of them were men (44.9%). Hypertension, diabetes, dyslipidemia, history of coronary heart disease, history of stroke, and atrial fibrillation (AF) were present in 85.7%, 56.4%, 58.6%, 41.1%, 16.2%, and 23.1%, respectively. The baseline vitals were as follows: body mass index (BMI), 24.1 ± 5.6 kg/m^2^; blood pressure (BP), 142.2 ± 27.7/82.3 ± 16.9 mmHg; mean BP, 102.2 ± 19.1 mmHg; initial pulse rate, 92.3 ± 20.6 beats per minute; initial RR, 24 breaths per minute; and LVEF, 45.4 ± 16.9%. All patients suffered from HF with NYHA class III (57.0%) and IV (42.9%). The baseline laboratory results were as follows: baseline NLR, 3.2 (IQR: 2.3, 5.0); MPV, 10.4 ± 0.9 fL; and MPVLR, 7.5 ± 4.9. The absolute lymphocyte count, MPV, NLR, and MPVLR are substantial predictors of CVEs.

### 3.2. Survival Analysis

**Figure 1:**
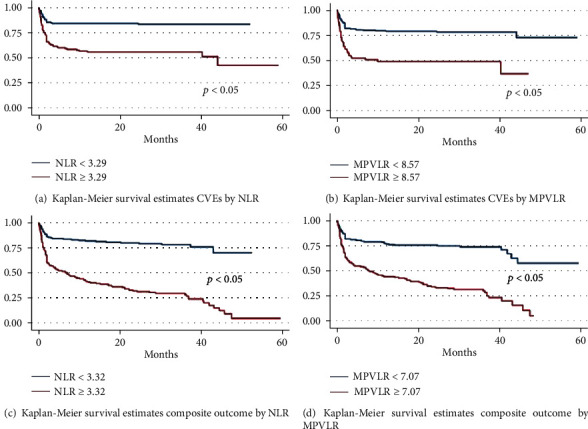
Kaplan-Meier survival estimation of NLR and MPVLR for cardiovascular events and a composite outcome.

Up to the end of the 3-year follow-up, 320 patients had a median follow-up time of 23 months (IQR: 2, 33 months). Of these, 96 patients (29.9%) had CVEs, 106 (33.0%) died, 62 (19.3%) were rehospitalized with HF, and 21 (6.5%) died at the time of admission. The incidence rate of CVEs was 60.57 per 1,000 population per year. Fifty percent of patients with HF were free of the composite outcome at approximately 39.8 months. The log-rank tests of equality across NLR and MPVLR groups for prediction of CVEs had a *P* value of <0.001 (Figures 1(a) and 1(b)); thus, the NLR and MPVLR groups were included as potential candidates for the final model. Tables 2 and 3 show that the univariate analyses using the Cox proportional hazard models were stratified by CVEs after HF inception. BMI; history of stroke; initial mean BP; initial RR; NYHA class IV; concomitant AF; ACS; valvular heart disease (VHD); prescribed angiotensin-converting enzymes inhibitors; and baseline NLR, MPV, MPVLR, and PLR were statistically significant covariates of CVE outcomes. In the multivariate analysis, initial RR, NYHA class IV, concomitant AF, ACS, and VHD were considerable variables with CVEs when the studied factors were NLR ≥ 3.29 (adjusted HR, 3.11; 95% CI, 1.98–4.89), MPVLR ≥ 8.57 (adjusted HR, 2.86; 95% CI, 1.87–4.39), or combined NLR and MPVLR (adjusted HR, 2.72; 95% CI, 2.15–3.43 (data not shown)).

For readmission for HF outcome, NYHA class IV, concomitant ACS, prescribed beta-blocker during hospitalization, and anemic status were significant variables in the multivariate Cox proportional hazard model (Table 3). The adjusted HR was 2.70 (95% CI, 1.58–4.61), 2.84 (95% CI,1.59–5.07), and 2.73 (95% CI, 1.93–3.85) for NLR ≥ 3.58, MPVLR ≥ 6.43, and combined NLR and MPVLR, respectively. NYHA class IV, initial systolic BP, and RR were significant factors associated with in-hospital mortality in patients with HF. The adjusted HRs were 9.54 (95% CI, 2.19–41.40), 7.87 (95% CI, 2.56–24.19), and 1.05 (95% CI, 1.03–1.07) for NLR ≥ 3.29, MPVLR ≥ 8.57, and combined NLR and MPVLR, respectively. In addition, NYHA class IV, concomitant AF, ACS, and BMI were significant variables associated with the composite outcome, with adjusted HRs of 4.76 (95% CI, 3.29–6.89), 3.64 (95% CI, 2.58–5.15), and 2.60 (95% CI, 2.17–3.12) for NLR ≥ 3.32, MPVLR ≥ 7.07, and combined NLR and MPVLR, respectively.

### 3.3. AUC Analyses of NLR, MPVLR, and Combined NLR and MPVLR for Each Outcome

**Figure 2:**
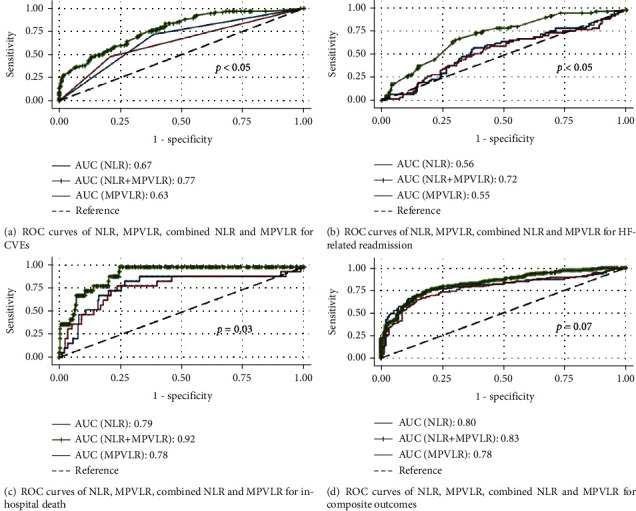
Receiver operating characteristics curves and AUC of NLR, MPVLR, and combined NLR and MPVLR for each outcome.

The receiver operating characteristic curves of NLR, MPVLR, and combined NLR and MPVLR for each outcome are illustrated in Figure 2. The AUC of NLR and MPVLR for CVEs were 0.67 (95% CI, 0.61–0.72) and 0.63 (95% CI, 0.58–0.69). Combining NLR and MPVLR increased the AUC value to 0.77 (95% CI, 0.72–0.83) with statistical significance. Likewise, combining NLR and MPVLR substantially strengthened the AUC value for predicting readmission for HF and in-hospital mortality (0.72 (95% CI, 0.65–0.79, *P* < 0.05) and 0.92 (95% CI, 0.88–0.96, *P* = 0.03). For the composite outcome, the AUC of NLR, MPVLR, and combined NLR and MPVLR were 0.80 (95% CI, 0.75–0.85), 0.78 (95% CI, 0.72–0.83), and 0.83 (95% CI, 0.79–0.88), respectively (Table 4). However, combining both the NLR and MPVLR did not significantly improve its performance in predicting the composite outcome (*P* = 0.07).

## 4. Discussion

This study evaluated the predictive role of the NLR and MPVLR on CVEs, readmission for HF, in-hospital death, and composite outcomes of patients hospitalized with AHF. During the 3-year follow-up, higher levels of NLR and MPVLR at baseline were independently associated with all outcomes after discharge. Notably, combining both NLR and MPVLR improved the ability to portend CVEs, readmission for HF, and in-hospital mortality than individual NLR or MPVLR.

The findings of this study correspond with the results from other published studies. In a previous prospective study of patients with acute decompensated HF, elevated NLR was associated with higher rates of in-hospital, 3-year mortality after discharge [22, 23], and heart transplantation risk [23]. A prior meta-analysis conducted by Wang et al. [10] also supported the predictive role of the NLR for all-cause mortality in patients with HF. All-cause mortality in this study includes both CV and non-CV deaths; however, no study has evaluated a particular outcome of CV mortality and CVEs in patients with AHF. Moreover, no study has investigated the prognostic role of NLR, MPVLR, and combined NLR and MPVLR for CV mortality and CVEs in adult patients hospitalized with AHF. Thus, this study was the first analysis of the capability of them, to predict an imperative CV outcome in patients with AHF.

The association between high NLR levels and worse CV outcomes in patients with HF could be explained by the activation of neutrophils from inflammatory and autonomic responses [24]. The release of proteolytic cytokines, such as acid phosphatase, elastase, and myeloperoxidase leads to an excess of free radicals which may cause myocardial injury. The neutrophil-derived enzyme myeloperoxidase has been studied in the context of ischemic myocardial damage [25, 26] and advanced HF [27], which is a potential mediator of impaired myocardial remodeling and poor prognosis. Furthermore, the elevation of ventricular filling pressure in patients with HF produces splanchnic congestion, which may affect the enteral loss of lymphocytes [28] and promote bacterial endotoxin translocation from the gut into the systemic circulation [29]. The levels of cortisol and tumor necrosis factor-1 during stress and immunologic response are also enhanced, which induces a diminished number of lymphocytes. Moreover, the release of cytokines, such as tumor necrosis factor-1, from the activation of the immunologic response may diminish lymphocyte counts [30]. The mouse model study supports the finding that T-cells can inhibit the development of intimal thickening and the proliferation of smooth muscle cells, endothelial cells, and fibroblasts by secretion of interferon-gamma [30]. Therefore, tissue healing is inhibited during the phase of T-cell activation and immune responses after injury.

In addition, MPV has been found to be an independent variable for predicting in-hospital and 6-month mortality [16] and HF-related hospitalization outcomes in patients with HF [17]. However, it was a good predictor of CVE, in-hospital death, and readmission for HF, but not for total mortality in our study. It has become evident that MPV is an indicator of both proinflammatory and prothrombotic conditions. The possible mechanism of MPV accompanying HF may be the MPV indicating the platelet size and function. The size of platelets in the circulation is correlated with the intensity of systemic inflammation [31]. Some genetic factors play a possible role in the regulation of MPV in inflammation and thrombosis [32]. Moreover, prior evidence derived from both retrospective and prospective studies proposes that a large platelet size and high MPV are predictors of established CV risk factors [31, 33] and thrombotic events in several CV and venous disorders, such as coronary artery disease, myocardial infarction, restenosis after percutaneous coronary intervention, cerebrovascular disease, AF, venous thromboembolism, and mortality [14, 15, 34]. Some biomarkers of inflammation are associated with HF [35], and platelet size is increased in both acute decompensated and chronic HF [16, 17, 36].

Our study had several strengths. This was the first to analyse the prognostic factors for CV outcomes in patients with AHF using routinely measured biomarkers (NLR, MPVLR, and combined NLR and MPVLR) by automated cell counters. The combination of the NLR and MPVLR had better performance for predicting CVEs and composite outcomes. A previous study reported a higher cut-off value of NLR to predict the mortality outcome compared to ours (NLR 5 to 7) [22], in which its performance was not reported. Additionally, HF is an endpoint of untreated CV disorders. As such, our study included those as confounding factors in the multivariate analysis, that is, concomitant AF, ACS, and significant VHD, and still found a significant association between the level of NLR and MPVLR, and CVEs. These results support the role of inflammation in the development and progression of different etiologies of HF. Therefore, NLR and MPVLR are potentially cost-effective biomarkers for the prediction of short- and long-term CVE outcomes and follow-up care of patients with HF.

This study had some limitations. This study was a retrospective cohort analysis that could not collect and evaluate the variation of NLR and MPVLR on clinical outcomes over a follow-up period. It was also based on a single center and was restricted to patients with AHF requiring hospitalization, which may have introduced bias. Our analysis did not include some potential confounders, such as concomitant inflammation, malignancy, and genetic factors, because of our limited data. Due to many missing data (86.3%), B-type natriuretic peptide and/or NT-pro-BNP were not analyzed in the study. However, a recent study revealed that the NLR was comparable with NT-pro-BNP as a prognostic marker in elderly patients with chronic HF [13]. Therefore, the varying cut-off values of NLR for HF outcomes between studies is a possible barrier to its application in clinical practice. Further studies are needed to explore the specific cut-off values of NLR and MPVLR for predicting HF outcomes in both acute and chronic HF settings.

Our findings demonstrated that 13.4% of patients with AHF had ACS as a precipitating factor, and the percentages of those with CVEs had ACS as their comorbidity was significantly higher than those without CVEs (Table 1). We also found ACS to be a significant confounding factor for CVE outcomes in the univariate and multivariate analyses. Similar to real-world data, AHF is a frequent consequence of ACS, which has an incidence of 6% to >45% [37], and their combination is associated with a poor prognosis [38]. Cardiac biomarkers of myocardial injury in patients with AHF in the absence of ACS are frequently raised and remain a diagnostic dilemma for those with ACS on admission; therefore, a diagnosis differentiating myocardial infarction and injury should be established in all patients with AHF [37]. Given our study's retrospective design, we were not able to confirm an associated ACS condition in all subjects with AHF. Further prospective studies focusing on ACS diagnosis of patients with AHF and prognostic differences between those with ACS on and during admission are also needed. Lastly, the new universal definition and classification of HF have just been launched [38], and additional studies on patients with HF with improved ejection fraction should be conducted.

## 5. Conclusion

Our study demonstrated that elevated NLR and MPVLR on admission in patients with AHF were independently associated with worse CVEs, rehospitalization for HF, in-hospital death, and composite outcomes of CVEs and all-cause mortality. NLR ≥ 3.29 and MPVLR ≥ 8.57 were also significant predictors for CVEs and in-hospital mortality. Interestingly, combining both NLR and MPVLR had superior performance compared to individual NLR or MPVLR. These economic biomarkers should be considered in the management and follow-up care of patients with AHF. Based on our single-center, retrospective cohort study, we recommend exploring this relationship and determining the optimal cut-off values of NLR and MPVLR for patients with both acute and chronic HF using other well-designed datasets.

## Figures and Tables

**Table 1:** Baseline characteristics of patients with heart failure, stratified by cardiovascular events.

Characteristics	Mean (SD)	CVEs (*N* = 96)	No CVEs (*N* = 225)	*z*/*X*^2^	*P* value^a^
Male sex, *N* (%)	144 (44.9)	40 (41.7)	104 (46.2)	-0.62	0.53
Age (years)	67.4 (14.9)	69.5 (13.6)	66.5 (15.4)	1.80	0.07
BW (kg)	61.7 (15.9)	58.4 (12.2)	63.2 (17.2)	2.48	0.01
Height (cm)	159.7 (8.9)	159.9 (8.9)	159.7 (8.9)	-0.24	0.81
BMI (kg/m^2^)	24.1 (5.6)	22.8 (4.3)	24.7 (5.9)	-2.70	<0.05
BMI class, *N* (%)					
<18.5 kg/m^2^	33 (10.3)	12 (12.5)	21 (9.3)		
18.5-24.9 kg/m^2^	172 (53.6)	60 (62.5)	112 (49.8)	7.56	0.06
25.0-29.9 kg/m^2^	79 (24.6)	16 (16.7)	63 (28.0)		
30.0 kg/m^2^	37 (11.5)	8 (8.3)	29 (12.9)		
Hypertension, *N* (%)	275 (85.7)	79 (82.3)	196 (87.1)	-1.11	0.27
Diabetes mellitus, *N* (%)	181 (56.4)	52 (54.2)	129 (57.3)	-0.28	0.78
Dyslipidemia, *N* (%)	188 (58.6)	56 (58.3)	132 (58.7)	0.03	0.98
Coronary heart disease, *N* (%)	132 (41.1)	73 (55.3)	59 (44.7)	1.69	0.09
Cerebrovascular accident, *N* (%)	52 (16.2)	24 (25.0)	28 (12.4)	2.81	<0.05
CKD stage, *N* (%)					
Stage 1	166 (51.7)	49 (51.0)	117 (52.0)		
Stage 2	10 (3.1)	3 (3.1)	7 (3.1)		
Stage 3	50 (15.6)	14 (14.6)	36 (16.0)	3.20	0.53
Stage 4	32 (9.9)	14 (14.6)	18 (8.0)		
Stage 5	63 (19.6)	16 (16.7)	47 (20.9)		
Alcohol consumption^∗^, *N* (%)					
Never	247 (82.1)	79 (84.9)	168 (80.8)		
Quit	35 (11.6)	10 (10.8)	25 (12.0)	0.96	0.62
Current	19 (6.3)	4 (4.3)	15 (7.2)		
Smoking^∗^, *N* (%)					
Never	223 (73.4)	75 (79.8)	148 (70.5)		
Quit	59 (19.4)	15 (15.9)	44 (20.9)	3.89	0.14
Current	22 (7.2)	4 (4.3)	18 (8.6)		
Rhythm, *N* (%)					
Sinus	247 (76.9)	64 (66.7)	183 (81.3)	7.66	<0.05
Atrial fibrillation	74 (23.1)	32 (33.3)	42 (18.7)		
LVEF (%)	45.4 (16.9)	45.2 (16.9)	45.5 (17.0)	-0.07	0.94
Type of HF, *N* (%)					
HFrEF	127 (39.6)	37 (38.5)	90 (40.0)	0.03	0.87
HFmEF	39 (12.2)	13 (13.5)	26 (11.6)	0.19	0.66
HFpEF	155 (48.3)	46 (47.9)	82 (48.4)	0.00	0.96
SBP (mmHg)	142.2 (27.7)	138.6 (28.2)	143.7 (27.4)	-1.79	0.07
DBP (mmHg)	82.3 (16.9)	80.0 (18.3)	83.2 (16.3)	-1.85	0.06
Mean BP (mmHg)	102.2 (19.1)	99.6 (19.8)	103.4 (18.7)	-1.96	0.05
Pulse rate (bpm)	92.3 (20.6)	93.4 (25.4)	91.8 (18.3)	0.87	0.38
Respiratory rate (per min)	23.6 (4.3)	24.5 (4.3)	23.2 (4.2)	2.77	<0.05
NYHA classification, *N* (%)					
III	183 (57.0)	39 (40.6)	144 (64.0)	3.99	<0.05
IV	138 (42.9)	57 (59.4)	81 (36.0)		
Comorbidity, *N* (%)					
Acute coronary syndrome	43 (13.4)	22 (22.9)	21 (9.3)	3.13	<0.05
Valvular heart disease^b^	57 (17.8)	25 (26.0)	32 (14.2)	2.41	0.02
Cardiomyopathy (CM)	181 (56.4)	55 (57.3)	126 (56.0)		
Ischemic CM	87 (27.1)	31 (32.3)	56 (24.9)		
Nonischemic CM	50 (15.6)	11 (11.5)	39 (17.3)	2.79	0.41
Hypertensive CM	44 (13.7)	13 (13.5)	31 (13.8)		
HT emergency	32 (9.9)	7 (7.3)	25 (11.1)	-1.11	0.27
Medication, *N* (%)					
Statin	235 (73.2)	75 (78.1)	160 (71.1)	1.19	0.23
Beta-blocker	182 (56.7)	60 (62.5)	122 (54.2)	1.19	0.24
ACEIs	101 (31.5)	22 (22.9)	79 (35.1)	-2.19	0.03
ARBs	55 (17.1)	20 (20.8)	35 (15.6)	1.26	0.21
Aspirin	177 (55.1)	54 (56.3)	123 (54.7)	0.33	0.74
P2Y12 inhibitors	92 (28.7)	32 (33.3)	60 (26.7)	2.18	0.34
Diuretics	224 (69.8)	60 (62.5)	164 (72.9)	-1.90	0.06
*Lab*					
WBC, ×10^3^, median (IQR) (cells/mL)	8.20 (6.4, 10.4)	8.6 (6.7, 10.4)	8.1 (6.4, 10.0)	-0.66	0.51
Neutrophil (%)	68.0 (11.0)	71.6 (10.7)	66.5 (10.8)	3.89	<0.001
Neutrophil count, ×10^3^, median (IQR) (cells/mL)	5.4 (4.3, 7.1)	6.1 (4.6, 7.4)	5.1 (4.0, 7.0)	-0.36	0.72
Lymphocyte (%)	22.5 (10.6)	19.2 (9.9)	23.9 (10.6)	-3.87	<0.001
Lymphocyte count, ×10^3^, median (IQR) (cells/mL)	1.7 (1.1, 2.4)	1.3 (0.9, 2.0)	1.8 (1.2, 2.4)	-2.07	0.04
NLR, median (IQR)	3.2 (2.3, 5.0)	5.0 (3.4)	3.6 (2.7)	4.25	<0.001
Q1 (NLR < 3.29)	2.2 (0.7)	1.9 (0.7)	2.3 (0.7)	5.24	<0.001
Q2 (NLR ≥ 3.29)	5.9 (3.3)	6.2 (3.3)	5.8 (3.3)		
Platelets, ×103 (cells/mL)	254.7 (92.4)	251.3 (94.5)	256.1 (91.6)	-0.50	0.61
Hb (g/dL)	10.7 (2.6)	10.7 (2.5)	10.7 (2.7)	-0.08	0.94
Anemia^c^	245 (76.3)	74 (77.1)	171 (76.0)	0.32	0.75
MPV (fL)^∗^	10.4 (0.9)	10.7 (1.0)	10.3 (0.9)	2.80	0.005
MPVLR^∗^	7.5 (4.9)	9.0 (5.7)	6.9 (4.5)	3.92	<0.001
Q1 (MPVLR < 8.57)	5.0 (1.9)	5.1 (2.0)	5.0 (1.8)	5.12	<0.001
Q2 (MPVLR ≥ 8.57)	13.6 (4.9)	13.4 (5.3)	13.9 (4.6)		
BUN, median (IQR) (mg/dL)	24.9 (18.1, 43.5)	25.5 (18.8, 40.5)	24.6 (16.9, 43.9)	-0.15	0.89
Creatinine, median (IQR), mg/dL	1.5 (1.1, 2.7)	1.5 (1.1, 2.6)	1.5 (1.0, 2.8)	-0.23	0.82
Sodium (mmol/L)	136.2 (8.0)	136.4 (4.0)	136.2 (9.2)	0.17	0.86
Potassium, median (IQR) (mmol/L)	4.0 (3.6, 4.4)	4.0 (3.6, 4.4)	4.0 (3.6, 4.5)	-0.36	0.72
Magnesium (mg/dL)^∗^	2.1 (0.4)	2.0 (0.5)	2.1 (0.3)	-0.13	0.89
Phosphorous (mmol/L)^∗^	4.1 (1.3)	4.3 (1.4)	4.1 (1.2)	0.64	0.52
Albumin (g/dL)^∗^	3.5 (0.5)	3.4 (0.5)	3.5 (0.6)	-1.10	0.27
Troponin, median (IQR), (ng/mL)^∗^	57.7 (20.0, 172.0)	115.0 (55.0, 410.5)	40.0 (16.0, 137.0)	1.47	0.14
NT-pro-BNP (×10^3^), median (IQR) (pg/mL)^∗^	5.2 (2.6-13.6)	9.9 (4.5, 14.1)	4.2 (1.7, 12.7)	0.92	0.36

Data are reported by mean ± SD or count (%); IQR: interquartile range. Abbreviations: CVEs: cardiovascular events; BW: body weight; BMI: body mass index; CKD: chronic kidney disease; LVEF: left ventricular ejection fraction; HFrEF: heart failure with reduced ejection fraction; HFmEF: heart failure with midrange ejection fraction; HFpEF: heart failure with preserved ejection fraction; SBP: systolic blood pressure; DBP: diastolic blood pressure; NYHA: the New York Heart Association Functional Classification; ACEIs: angiotensin-converting enzyme inhibitors; ARBs: angiotensin-II receptor antagonists; WBC: white blood cell; NLR: neutrophil-lymphocyte ratio; Hb: hemoglobin; MPV: mean platelet volume; MPVLR: MPV-to-lymphocyte ratio; NT-pro-BNP: N-terminal pro-B-type natriuretic peptide. ^∗^Missing data: alcohol consumption = 6.2%, smoking = 5.3%, MPV and MPVLR = 5.9%, magnesium = 51.7%, phosphorous = 57%, albumin = 42.1%, troponin = 50.8%, and NT − pro − BNP = 86.3%. ^a^Using *t*-test for continuous variables and *χ*^2^ test for categorical variables. ^b^Valvular heart disease (VHD): mitral valve disease (5.3%), aortic valve disease (9.9%), others (2.5%). ^c^Anemia: Hb < 13 g/dL in men and Hb < 12 g/dL in women.

**Table 2:** Univariate and multivariate analysis with a Cox proportional hazard model of risk factors for cardiovascular events of patients with heart failure.

Variables	Univariate analysis						Multivariate analysis (NLR)				Multivariate analysis (MPVLR)				Multivariate analysis (NLR and MPVLR)			
	*β*	HR	*Z*	95% CI		*P* value	HR	95% CI		*P* value	HR	95% CI		*P* value	HR	95% CI		*P* value
				Lower	Upper			Lower	Upper			Lower	Upper			Lower	Upper	
NLR																		
NLR < 3.29	1	1				<0.05	1			<0.05					1			<0.05
NLR ≥ 3.29	1.19	3.30	5.24	2.11	5.16		3.11	1.98	4.89						2.42	1.46	4.01	
MPVLR																		
MPVLR < 8.57	1					<0.05					1			<0.05	1			<0.05
MPVLR ≥ 8.57	1.08	2.93	5.12	1.94	4.42						2.86	1.87	4.39		1.95	1.22	3.10	
BMI	-0.06	0.94	-2.70	0.90	0.98	<0.05												
CVA	0.67	1.95	2.81	1.22	3.10	<0.05												
Rhythm																		
Sinus	1	1				<0.05	1			0.02	1			<0.05	1			0.05
Atrial fibrillation	0.63	1.88	2.89	1.22	2.89		1.76	1.12	2.78		2.86	1.87	4.39		1.60	1.00	2.56	
Mean BP	-0.01	0.99	-1.96	0.98	1.00	0.05												
Respiratory rate	0.05	1.06	2.77	1.02	1.09	<0.05	1.05	1.00	1.09	0.04	1.05	1.00	1.09	0.03	1.05	1.00	1.09	0.04
NYHA																		
III	1	1				<0.05	1				1			<0.05	1			<0.05
IV	0.84	2.32	3.99	1.53	3.49		2.21	1.45	3.38	<0.05	2.14	1.39	3.31		2.16	1.40	3.34	
Etiology																		
ACS	0.77	2.17	3.13	1.33	3.52	<0.05	2.17	1.32	3.57	<0.05	2.41	1.46	3.96	<0.05	2.33	1.41	3.86	<0.05
VHD	0.57	1.77	2.41	1.11	2.81	0.02	1.71	1.06	2.75	0.03	1.71	1.04	2.79	0.03	1.83	1.12	2.98	0.02
Medication																		
ACEIs	-0.54	0.58	-2.19	0.36	0.94	0.03												
PLR	0.002	1.00	3.09	1.001	1.003	<0.05												
MPV	0.27	1.31	2.80	1.08	1.58	<0.05												

Abbreviations: NLR: neutrophil-lymphocyte ratio; MPVLR: mean platelet volume-to-lymphocyte ratio; CI: confidential interval; HR: hazard ratio; BMI: body mass index; CVA: cerebrovascular accident; BP: blood pressure; NYHA: New York Heart Association Classification; ACS: acute coronary syndrome; VHD: valvular heart disease; ACEIs: angiotensin-converting enzyme inhibitors; PLR: platelet to lymphocyte ratio; MPV: mean platelet volume.

**Table 3:** Association of NLR and MPVLR with a prognosis of subsequent cardiovascular events and mortality in patients with heart failure.

Outcome	Number of events	Crude HR	95% CI	*P* value	Adjusted HR	95% CI	*P* value
Cardiovascular events^a^							
NLR < 3.29	27	1 (reference)	1 (reference)	<0.001	1 (reference)	1 (reference)	<0.001
NLR ≥ 3.29	69	3.30	2.11-5.16		3.11	1.98-4.89	
MPVLR < 8.57	48	1 (reference)	1 (reference)	<0.001	1 (reference)	1 (reference)	<0.001
MPVLR ≥ 8.57	44	2.93	1.94-4.42		2.86	1.87-4.39	
Rehospitalization for HF^b^							
NLR < 3.58	26	1 (reference)	1 (reference)	0.002	1 (reference)	1 (reference)	<0.001
NLR ≥ 3.58	36	2.23	1.34-3.69		2.70	1.58-4.61	
MPVLR < 6.43	25	1 (reference)	1 (reference)	0.020	1 (reference)	1 (reference)	<0.001
MPVLR ≥ 6.43	35	1.84	1.10-3.08		2.84	1.59-5.07	
In-hospital death^c^							
NLR < 3.29	3	1 (reference)	1 (reference)	0.002	1 (reference)	1 (reference)	0.003
NLR ≥ 3.29	18	10.62	2.46-45.81		9.54	2.19-41.40	
MPVLR < 8.57	4	1 (reference)	1 (reference)	<0.001	1 (reference)	1 (reference)	<0.001
MPVLR ≥ 8.57	15	10.43	3.45-31.55		7.87	2.56-24.19	
Composite outcome^d^							
NLR < 3.32	38	1 (reference)	1 (reference)	<0.001	1 (reference)	1 (reference)	<0.001
NLR ≥ 3.32	119	4.82	3.34-6.96		4.76	3.29-6.89	
MPVLR < 7.07	50	1 (reference)	1 (reference)	<0.001	1 (reference)	1 (reference)	<0.001
MPVLR ≥ 7.07	100	3.50	2.49-4.92		3.64	2.58-5.15	

Abbreviations: NLR: neutrophil-lymphocyte ratio; MPVLR: mean platelet volume-to-lymphocyte ratio; CI: confidential interval; HR: hazard ratio; HF: heart failure; NYHA: New York Heart Association; ACS: acute coronary syndrome. ^a^A multivariate Cox proportional hazard model adjusted for NYHA classification, ACS, heart rhythm, initial respiratory rate, and underlying valvular heart disease (VHD). ^b^A multivariate Cox proportional hazard model adjusted for NYHA classification, ACS, taking beta-blocker, and anemia. ^c^A multivariate Cox proportional hazard model adjusted for NYHA classification, initial systolic blood pressure, and initial respiratory rate. ^d^A multivariate Cox proportional hazard model adjusted for NYHA classification, ACS, heart rhythm, and body mass index.

**Table 4:** Area under the receiver operating characteristics curve (AUC) stratified according to each outcome.

Variables	AUC	95% CI	Sensitivity (%)	Specificity (%)	*P* value
Cardiovascular events					
NLR	0.67	0.61-0.72	75.2	66.1	<0.05
MPVLR	0.63	0.58-0.69	45.3	79.7	
Combined NLR and MPVLR	0.77	0.72-0.83	84.6	58.7	
Rehospitalization					
NLR	0.56	0.48-0.64	61.2	61.0	<0.05
MPVLR	0.55	0.46-0.63	60.2	57.1	
Combined NLR and MPVLR	0.72	0.65-0.79	82.1	60.8	
In-hospital mortality					
NLR	0.79	0.66-0.91	87.5	70.8	0.03
MPVLR	0.78	0.65-0.90	79.6	75.7	
Combined NLR and MPVLR	0.92	0.88-0.96	88.7	85.5	
Composite outcome					
NLR	0.80	0.75-0.85	71.6	86.8	0.07
MPVLR	0.78	0.72-0.83	62.8	91.1	
Combined NLR and MPVLR	0.83	0.79-0.88	79.8	84.7	

Abbreviations: NLR: neutrophil-lymphocyte ratio; MPVLR: mean platelet volume-to-lymphocyte ratio; CI: confidential interval.

## Data Availability

The data used to support the findings of this study are included within the article.

## Appendix

### Calculation of Combined NLR and MPVLR

Combined NLR and MPVLR was derived from *β*-coefficients of the final Cox regression multivariate model of the individual outcome as shown below (abbreviations: NLR: neutrophil-lymphocyte ratio; MPVLR: mean platelet volume-to- lymphocyte ratio; NYHA: New York Heart Association; ACS: Acute coronary syndrome; VHD: valvular heart disease; RR: respiratory rate; SBP: systolic blood pressure; BMI: body mass index).

- *C*_CVE_ (combined NLR and MPVLR of CVE) = [(0.89 × NLR) + (0.67 × MPVLR) + (0.77 × NYHA) + (0.85 × ACS) + (0.60 × VHD) + (0.47 × rhythm) + (0.05 × RR)]
- *C*_HF rehospitalization_ (combined NLR and MPVLR of HF rehospitalization) = [(0.67 × NLR) + (0.68 × MPVLR) + (0.58 × NYHA) + (1.05 × ACS) + (0.77 × BetaBlocker use) − (0.79 × anemia)]
- *C*_InhospDeath_ (combined NLR and MPVLR of in − hospital death) = [(1.62 × NLR) + (1.51 × MPVLR) + (2.05 × NYHA) − (0.03 × SBP) + (0.14 × RR)]
- *C*_Composite_ (combined NLR and MPVLR of composite outcome) = [(1.22 × NLR) + (0.76 × MPVLR) + (0.65 × NYHA) + (0.52 × ACS) + (0.36 × rhythm) − (0.04 × BMI)]

For the categorical variables of ACS, VHD, BetaBlocker use, and anemia, the patients were assigned the value 1 for the category they belong to and 0 for the other categories, whereas those with variables of NYHA class IV, AF rhythm, NLR, and MPVLR equal or above cut-offs were designated as 1 and 0 for the others. Different cut-off values for categorical NLR and MPVLR of each outcome are as follows: NLR = 3.29 and MPVLR = 8.57 for CVEs and in-hospital death outcomes; NLR = 3.58 and MPVLR = 6.43 for rehospitalization for HF; and NLR = 3.32 and MPVLR = 7.07 for the composite outcome.
